# Necl-4/SynCAM-4 Is Expressed in Myelinating Oligodendrocytes but Not Required for Axonal Myelination

**DOI:** 10.1371/journal.pone.0064264

**Published:** 2013-05-20

**Authors:** Ying Zhu, Hong Li, Kehan Li, Xiaofeng Zhao, Tai An, Xuemei Hu, Jinsil Park, Hao Huang, Yin Bin, Boqin Qiang, Jiangang Yuan, Xiaozhong Peng, Mengsheng Qiu

**Affiliations:** 1 Department of Anatomical Sciences and Neurobiology, University of Louisville School of Medicine, Louisville, Kentucky, United States of America; 2 The National Laboratory of Medical Molecular Biology, Institute of Basic Medical Sciences, Chinese Academy of Medical Sciences and Peking Union Medical College, Beijing, China; 3 Institute of Developmental and Regenerative Biology, College of Life and Environmental Sciences, Hangzhou Normal University, Hangzhou, China; Emory University, United States of America

## Abstract

The timing and progression of axonal myelination are precisely controlled by intercellular interactions between neurons and glia in development. Previous *in vitro* studies demonstrated that Nectin like 4 (Necl-4, also known as cell adhesion molecule Cadm-4 or SynCAM-4) plays an essential role in axonal myelination by Schwann cells in the peripheral nervous system (PNS). However, the role of Necl-4 protein in axonal myelination in the developing central nervous system (CNS) has remained unknown. In this study, we discovered upregulation of *Necl-4* expression in mature oligodendrocytes at perinatal stages when axons undergo active myelination. We generated *Necl4* gene knockout mice, but found that disruption of *Necl-4* gene did not affect oligodendrocyte differentiation and myelin formation in the CNS. Surprisingly, disruption of Necl-4 had no significant effect on axonal myelination in the PNS either. Therefore, our results demonstrated that *Necl-4* is dispensable for axonal myelination in the developing nervous system.

## Introduction

In vertebrate nervous system, internodal axons are wrapped by compact myelin sheaths, the specialized cellular membranes elaborated by myelinating glial cells. As myelin sheaths provide insulation for axons, action potentials propagate from node (of Ranvier) to node, and this saltatory conduction mechanism dramatically increases the transmission velocity of electrical impulses.

In the central nervous system (CNS), myelin sheaths are formed by oligodendrocytes. During development, oligodendrocytes originate from the neuroepithelium of the ventricular zone and then migrate to the surrounding white matter regions [1]–[3], where they contact target axons and subsequently differentiate into mature myelinating oligodendrocytes. The progression of axonal myelination involves multiple steps, including adherence of oligodendrocytes to axons, spiraling of oligodendrocyte process around axons and the formation of compact myelin sheath [4]. Each of these steps is precisely regulated by the reciprocal communication between glial cells and neurons [4], [5].

The molecular mechanisms that mediate the axonal-glial interaction and myelin formation in the CNS remain elusive. Recently, it was reported that cell adhesion molecules of the nectin-like (Necl) family are likely to be involved in axonal myelination process [6], [7]. The NECL proteins belong to the immunoglobin(Ig)-like CAM superfamily and contain three extracellular domains, a single transmembrane domain and a cytoplasmic domain with characteristic FERM- and class II PDZ-binding motifs [8]–[11]. Through their homophilic or heterophilc interactions, NECL proteins regulate a wide spectrum of biological processes including cell adhesion, cell proliferation, synapse assembly, and myelin formation [12], [13]. In the PNS, neurons express *Necl-1, Necl-2, Necl-4* and a low level of *Necl-3*, whereas Schwann cells only express *Necl-2* and *Necl-4*. Notably, *Necl-1* and *Necl-4* are located on the apposing sides of axonal-glial contact interface along the internodal region, with *Necl-1* on the axonal membrane and *Necl-4* on the glial membrane [6], [7]. There is a strong heterophilic interaction between *NECL-1* and *NECL-4* [12]. Disruption of Necl-4 expression or its interaction with Necl-1 abolished axonal myelination of dorsal root ganglion (DRG) neurons by Schwann cells in culture [6], [7], suggesting the critical role of Necl-4 in mediating axonal-glial interaction and PNS myelination.

However, it remains unknown whether *Necl-4* has a similar role in axonal myelination in the developing CNS, and whether it is required for PNS myelination *in vivo*. In this study, we showed that *Necl-4* is expressed in both CNS neurons and myelinating oligodendrocytes at postnatal stages when axons undergo active myelination. However, disruption of *Necl-4* alone had little effects on myelin formation in either the CNS or the PNS.

## Materials and Methods

### *In Situ* RNA Hybridization and Double Labeling Experiments

Mouse spinal cord and brain tissues from postnatal stages were perfused and fixed in 4% paraformaldehyde in PBS at 4°C overnight. Following fixation, tissues were transferred to 20% sucrose in PBS overnight, embedded in OCT media, and then sectioned on a cryostat. For double labeling experiments, tissues were first subjected to RNA *in situ* hybridization (ISH) with *Necl4* (GenBank accession no. NM_001047107) riboprobe, followed by anti-Olig2, anti-APC or anti-NeuN immunohistochemical staining with ABC kit, respectively. Rabbit anti-Olig2 (a gift from Dr. Charles Stiles) was used at 1∶2,000; mouse anti-APC (Ab-7, Oncogene Inc, Cat# ab167994) at 1∶3,000; and mouse anti-NeuN (Chemicon Inc, Cat# MAB377) at 1∶4,000.

### Generation of Necl-4 mutant mice

The BAC clone containing the genomic DNA of *Necl-4* was purchased from Invitrogen. The gene target vector was constructed by replacing the first exon with inducible Cre recombinase gene (Cre-ERT2) and the neomycin resistance gene. Linearized targeting vector was electroporated into mouse ES cells. Following selections, the genomic DNA of ES clones was digested with SpeI and subjected to Southern hybridization using 3′ flanking probe. The wild type allele yields a band of 8.9 kb and the mutant allele a band of 7.3 kb. 198 independent ES clones were screened by Southern blot genotyping with the 3′ flanking probe. Five clones with homologous recombination were identified and two were injected into blastocysts to produce chimera mice for germline transmission to produce the F1 heterozygous mice. The homozygous mutant animals derived from two independent ES clones exhibited the same phenotype. Germline transmission was confirmed by both Southern hybridization and PCR. The primers N4 neo-UP (5′ CGTTGGCTACCCGTGATATTGCTGAAGAGC) and N4 DP (5′- GGGACAAAGGCGGCGTGGAGAAACG-3) were to detect the mutant allele ( 1150 bp); PCR conditions were 95°C for 5 min; 35 cycles of 95°C for 30 s, 60°C for 45 s, 72°C for 1 min 20 sec, followed by incubation at 72°C for 10 min. The primers N4 WT-UP (5′ GCGGAGCAGAGGGCGGGACTGGACT -3′) and N4 DP (5′- GGGACAAAGGCGGCGTGGAGAAACG-3) were used to detect the wild type allele (725 bp); PCR conditions were 95°C for 5 min; 35 cycles of 94°C for 30 s, 63°C for 30 sec, 72°C for 45 sec, followed by incubation at 72°C for 10 min.

### Genotyping of *Nkx2.2* and *Olig1* mutant mice

All of the mice used in this study were handled according to the protocols approved by Institutional Animal Care and Use Committee (IACUC), University of Louisville (IACUC: 12034). The homozygous pups were obtained by interbreeding heterozygous animals. Genomic DNA extracted from tails was used for genotyping by Southern analysis or by PCR. Genotyping methods of *Olig1* and *Nkx2.2* loci were described earlier [14]–[16].

### RT-PCR

Total RNA was prepared from the brain of wild type and mutant mice at P7 with the RNA easy kit (Roche) and reverse-transcribed to cDNA with the first strand synthesis kit (Sigma). Primers Necl4Exon1UP 5′-GGG AGG TGC AGG TGC CGG G-3′ and Necl4Exon2DP 5′- GTG CCA TTG AAA AAG AGG GT -3′, which were respectively located in the first exon and the second exon, were designed to detect the 5′-end cDNA; Necl4Exon1UP 5′-GGG AGG TGC AGG TGC CGG G-3′ and Necl4 3-UTR DP 5′- CCA GGC ATC CAA CAC CC -3′, which were respectively located in the first exon and the 3′ end untranslated sequence, were used to detect the full length of cDNA. The PCR conditions were 95°C for 5 min; 30 cycles of 94°C for 30 s, 55°C for 30 sec, 72°C for 1 min, followed by incubation at 72°C for 10 min. GAPDH was the control.

### Western blotting

Brain tissues were lysed in tissue lysis buffer (Sigma) with protease inhibitor cocktail (Sigma). 30 mg protein from control and mutant tissues was loaded for SDS-PAGE electrophoresis and subsequently detected with anti-Necl-1 (developed in Peking Union Medical University), anti-Necl-2 (Proteintech, Cat# 14335-1-AP), anti-Necl-3 (Abcam Inc, Cat# ab133393) and anti-Necl-4 (UC Davis/NIH NeuroMab Facility, Cat#73-247), and mouse anti-β-actin (Sigma, Cat# A5316) antibodies according to the standard protocol. The integrated density of blots on films was assessed with the analysis tool in Adobe Photoshop CS5 software and the relative densitometric values were used for statistical analyses on the expression level of target proteins.

### Ultrastructural Analyses of Myelin Structures

Wild type and *Necl-4* mutant littermates were perfused with 3% glutaraldehyde in 0.1 M cacodylate buffer, pH 7.2, and small pieces of tissues from optic nerves, spinal cord (at T6 level), and sciatic nerves were removed and postfixed for three additional hours. Tissues were then washed several times with cacodylate buffer, postfixed in 1% osmium tetroxide for 1 h, washed again with the buffer before dehydration through a series of graded alcohol. Fixed tissues were subsequently embedded in epony plastic and sectioned at 800–1000 Å on a diamond knife and mounted on 200 mesh copper grids. Ultra-thin sections were stained with uranium acetate and lead citrate, and examined under a Philips CM12 EM operating at 80 kV. For statistical analyses of axonal myelination, three or four animals per each genotype were used, and the number or the perimeter of axons was counted with Adobe Photoshop CS5 software.

### Statistical analysis

Statistical analyses were performed with two-tailed homoscedastic (unpaired) Student's t-test. Error bars represented the standard deviations.

## Results

### Expression of Necl-4 in neurons and oligodendrocytes in the CNS

**Figure 1:**
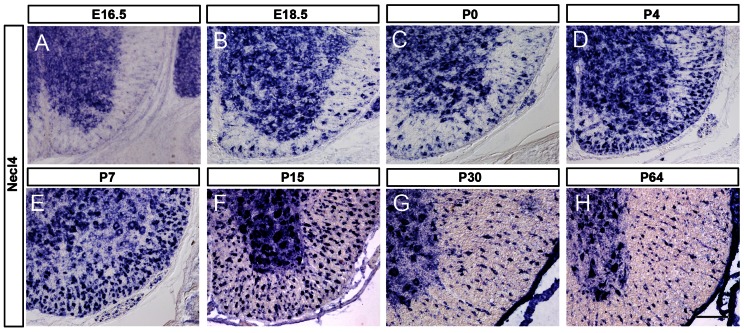
*Necl-4* expression in embryonic and postnatal spinal cords. **A–H:** Spinal cord sections from E16.5, E18.5, P0, P4, P7, P15, P30 and P64 were subjected to ISH with *Necl-4* riboprobe. Necl4 expression was detected in spinal cord white matter after E18.5, and persisted till adulthood. Scale bar, 100 µm.

Recent studies demonstrated that *Necl-4* is expressed in myelinating Schwann cells and is required for PNS myelination in cell co-culture [6], [7]. However, its expression and function in CNS development has remained unknown. To address this question, we first examined *Necl-4* expression in the developing mouse spinal cord tissue by RNA *in situ* hybridization (ISH). Prior to the onset of axonal myelination, *Necl-4* expression was restricted to the gray matter of spinal cord (Figure 1A). Starting from E18.5, expression of *Necl-4* was also detected in the white matter (Figure 1B), and the number of Necl-4+ cells gradually increased with time and reached the maximum between P7 and P15 (Figure 1E–F), the peak time of oligodendrocyte myelination in mouse spinal cords [4]. At later postnatal stages, *Necl-4* expression was gradually down-regulated (Figure 1G–H).

**Figure 2:**
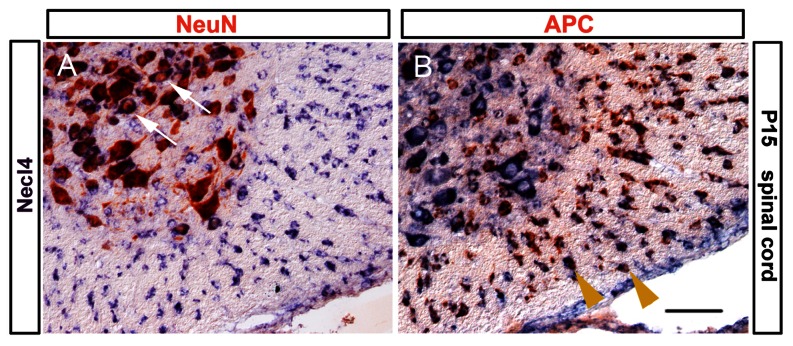
Co-expression of *Necl-4* with neuronal and oligodendroglial markers. Spinal cord sections from P15 were subjected to *Necl-4* ISH (in blue), followed by immunohistochemical staining for NeuN and APC, respectively. The arrows and arrowheads indicate representative double stained neurons and mature oligodendrocyte in the ventral white matter, respectively. Scale bar, 50 µm.

The spatiotemporal pattern of *Necl-4* expression in the developing spinal tissues suggested that *Necl-4* is first expressed in neurons, but later in mature myelinating oligodendrocytes. To examine this hypothesis, we carried out double staining with *Necl-4* ISH and immunohistochemical staining with anti-NeuN, or anti-APC antibodies in P7 and P15 spinal cord tissues. NeuN is a specific marker for postmitotic neurons [17] and APC/CC1 specifically marks mature differentiated oligodendrocytes [18], [19]. Double-staining experiments revealed numerous Necl-4+/NeuN+ neurons in the gray matter (Figure 2A). In the white matter, the vast majority of Necl-4+ cells co-expressed APC (Figure 2B). These results suggested that *Necl4* is selective expressed in neurons and mature oligodendrocytes.

**Figure 3:**
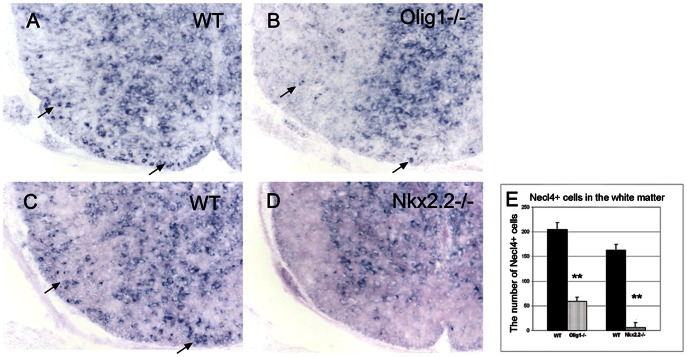
Reduced *Necl-4* expression in *Olig1* and *Nkx2.2* null mutant spinal cords. Spinal cord sections from wild-type, Olig1−/− and Nkx2.2 −/− mice at P3 were subjected to *Necl-4* ISH. As indicated by the arrow, *Necl-4* expression was significantly reduced in the white matter of mutant spinal cords. Scale bar, 100 µm. **E**. The counts of the Necl-4+ cells in the spinal cord white matter of wild type, Olig1 null mutant and Nkx2.2 null mutant mice. Student's t-test, n = 3, **p<0.01. Error bar, standard deviation.

To further confirm *Necl-4* expression in differentiated oligodendrocytes, we examined *Necl-4* expression in *Nkx2.2*−/− and *Olig1−/−* mutant mouse spinal cords by ISH. Previous studies demonstrated that oligodendrocyte differentiation was significantly delayed in both *Nkx2.2* and *Olig1* null mutant mice [15], [20]. Consistent with the notion that *Necl-4* is specifically expressed in differentiated oligodendrocytes, the number of Necl-4+ cells in the white matter of the spinal cord was significantly reduced in both *Olig1* and *Nkx2.2* mutants (Figure 3, *p*<0.01).

### Expression of Necl-4 in the brain

**Figure 4:**
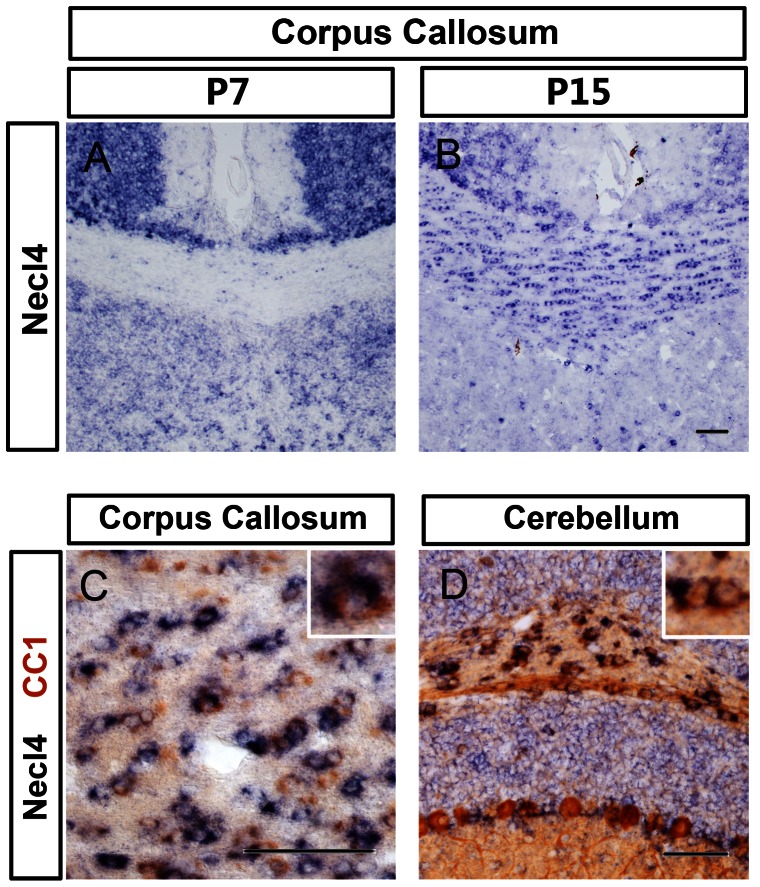
Expression of *Necl-4* in brain oligodendrocytes. **A–B**, *Necl-4* expression in corpus callosum as detected by ISH. **C–D**, The forebrain coronal sections (P15) and cerebellum sagittal sections (P7) were subjected to ISH with *Necl-4* probe (in blue) followed by immunohistochemical staining with anti-Olig2 antibody (in brown). The insets are the higher magnification of double positive cells. Scale bar, 50 µm.

To investigate whether there is a regional difference in *Necl-4* expression along the rostrocaudal axis, we carried out double-staining (ISH for *Necl4* and immunohistochemical staining for Olig2) in early postnatal brain tissues. Similar to our observations in the spinal cord, *Necl-4* expression in the forebrain was initially detected in neurons and later in oligodendrocytes. *Necl-4* expression in cerebral oligodendrocytes started to be detectable in corpus callosum at P7 (Figure 4A), but became more obvious at P15 (Figure 4B, C). In the cerebellum, *Necl-4* was strongly expressed in the gray matter at P7, especially the Purkinje cell layer, and in the white matter oligodendrocytes as well (Figure 4D). Later, strong expression of *Necl-4* was maintained in neurons, whereas its expression in the white matter glia was gradually down-regulated (data not shown). These results suggested that *Necl-4* is sequentially expressed in neurons and white matter oligodendrocytes in the rostral regions of the CNS as well, and its expression is also temporally coincident with the myelination process in the brain.

### Generation of *Necl-4* knockout mice

**Figure 5:**
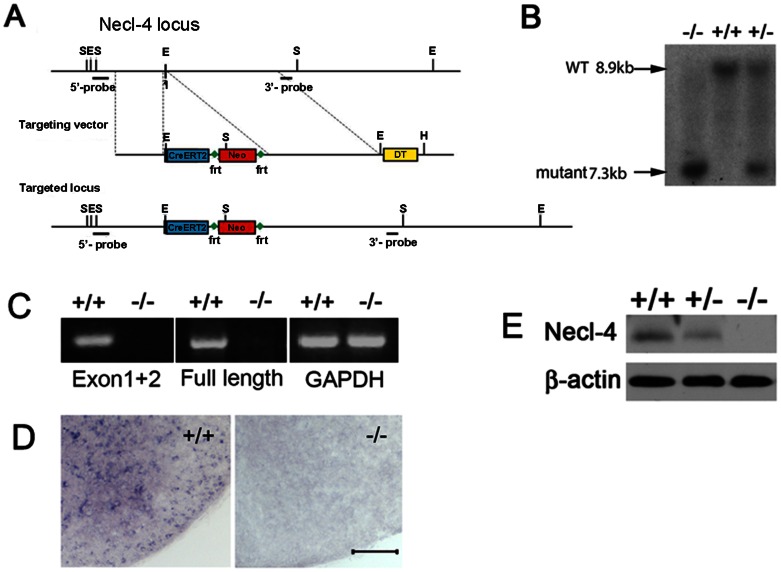
Generation of *Necl-4* knockout mice. **A.** Schematic representation of *Necl-4* gene-targeting vector. In the targeting vector, the first exon of *Necl-4* was replaced with the Cre-ERT2 and Neo cassette. **B.** Genotyping of F2 pups by Southern hybridization with 3′-probe. **C.** RT-PCR was performed to confirm the deletion of Necl-4 transcript. Primers were designed to detect transcription of the targeted 5′ end cDNA (exon 1 and exon2) and the full-length cDNA, respectively. GAPDH was the control. **D.** Spinal cord sections from WT and homozygous mice at P7 were subjected to *in situ* hybridization with Necl-4 riboprobe. Scale bar, 50 µm. **E.** Western immunoblotting of spinal cord tissues (from P7 wild-type, Necl-4 heterozygous and homozygous pups) with anti-Necl-4 monoclonal antibody. β-actin was used as the internal control.

To examine the *in vivo* role of *Necl-4* in axonal myelination, we constructed a gene-targeting vector to replace the first exon of *Necl-4* gene with the Neo cassette in embryonic stem (ES) cells by homologous recombination (Figure 5A). The first exon contains the only in-frame starting code (ATG) of the entire *Necl-4* coding sequence. Following electroporation and neomycin selection, two independent ES clones with homologous recombination were injected into blastocysts to produce chimera mice. Germ line transmission of the mutant allele in the offspring was confirmed by Southern blotting and PCR (Figure 5B and data not shown). Homozygous mice were viable after birth and morphologically indistinguishable from their littermates.

Disruption of *Necl-4* expression was confirmed by several molecular and biochemical approaches. RT-PCR and *in situ* hybridization were performed to detect *Necl-4* transcription. Two pairs of primers were designed to detect the 5′- transcript (including the targeted exon) and the full open reading frame (ORF), respectively. The results indicated that Necl-4 transcription is disrupted in the null mutants (Figure 5C). Consistently, *in situ* hybridization with Necl-4 probe in spinal cord sections could not detect the mRNA transcription of *Necl-4* gene in the N4−/− mice (Figure 5D), indicating the lack of Necl4 transcription in the null mutants. Western immunoblotting with anti-Necl-4 antibody revealed that the expression of NECL-4 protein was reduced in *Necl-4* heterozygous tissues and completely absent in the homozygous mutants (Figure 5E). These results suggested that the expression of *Necl4* was successfully deleted in *Necl4* homozygous mutants.

### Normal differentiation of oligodendroytes in Necl-4 mutant spinal cord

**Figure 6:**
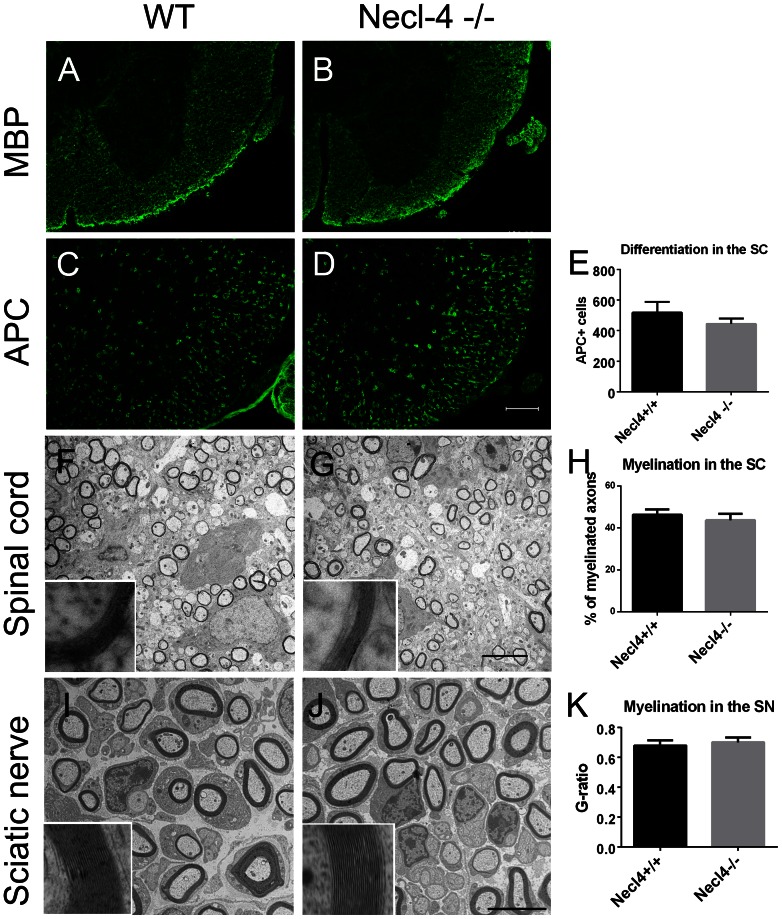
Oligodendrocyte differentiation and axonal myelination in *Necl4* null mutant mice. **A-D.** Immunofluorescent staining of wildtype and Necl-4−/− spinal cord tissues at P15 with mature markers MBP and APC. Scale bar, 100 µm. **E.** Statistical analysis of the number of APC+ cells at P15 (n = 4). Student's t-test, *p* = 0.11. Error bar, standard deviation. **F–K.** Myelination in spinal cord and sciatic nerves at P7 from the wild type and Necl-4 null mutants was examined under electron microscope. Scale bars, 5 µm. High magnification images of individual axons were shown as the inserts. **H.** Statistical analysis of the percentage of myelinated axons (n = 3). Student's t-test, *p* = 0.34. Error bar, standard deviation. **K.** Statistical analysis of the G-ratio of sciatic nerves (n = 3). Student's t-test, *p* = 0.39. Error bar, standard deviation. SC, spinal cord. SN, sciatic nerve.

Previous studies showed that knockdown of Necl-4 in Schwann cells and DRG co-culture resulted in loss of myelin gene expression and diminished expression of two transcription factors, Oct-6 and Krox-20, which are required for Schwann cell differentiation [6], [7]. As Necl-4 started to be expressed in differentiated oligodendrocytes at early perinatal stages, it may be required for oligodendrocyte differentiation. Immunostaining with antibodies against mature oligodendrocyte markers APC and MBP was performed to examine differentiation in oligodendrocytes. The results revealed a similar expression pattern in the wild-type and the Necl4 null mutant spinal cord tissues at P7 and P15 (Figure 6A–E and data not shown). The number of APC+ cells in the ventral white matter was counted, and there was no significant difference between the controls (518±70) and the mutants (444±35) (Figure 6E, *p* = 0.11). These results suggested that oligodendrocytes differentiate normally in the *Necl4* null mutants.

### Normal axonal myelination in *Necl-4* knockout

We next investigated whether Necl-4 is required in the CNS myelination by examining myelin structures in the spinal cord at various postnatal developmental stages. At P7, a large number of axons were myelinated in the ventral white matter (at the position of corticospinal tract at T6) (Figure 6F–G). The density of axons in the mutants (304,157±25,470/mm^2^) was comparable with that in the controls (309,145±22,474/mm^2^) (Figure S1A, n = 3, *p* = 0.81). The myelin structures were found normal in *Necl-4* mutants, and the percentage of myelinated axons in the mutant mice (43.74±3.04%) was slightly lower, but not significantly different from that in the wild-type littermates (46.25±2.55%) (Figure 6H, *p* = 0.34). Therefore, CNS myelination was not significantly affected by the *Necl-4* mutation.

Recent studies suggested that reciprocal interaction between NECL-4 and NECL-1 was essential for the ensheathment and myelin wrapping of axons by Schwann cells in the DRG-Schwann cell co-culture [6], [7]. Therefore, we also examined the PNS myelination in sciatic nerves at P7. The g-ratio (the ratio of axon diameter to the diameter of axon and myelin sheath) was calculated to analyze the thickness of myelin sheaths in sciatic nerves. The densities of myelinated axons were comparable between the controls (57,412±4,992/mm^2^) and mutants (50,903±14,464/mm^2^) (Figure S1B). The average thickness of myelin sheath of Necl-4−/− (g-ratio = 0.70±0.03) was slightly, but not significantly thinner than that of the controls (g-ratio = 0.68±0.04) (Figure 6I–K, *p* = 0.39). These results indicated that myelination proceeded normally in the Necl4−/− sciatic nerves. Therefore, *Necl-4* appears to be dispensable for the PNS myelination by Schwann cells during *in vivo* development.

### Lack of functional compensation between Necl4 and of other Necl molecules

**Figure 7:**
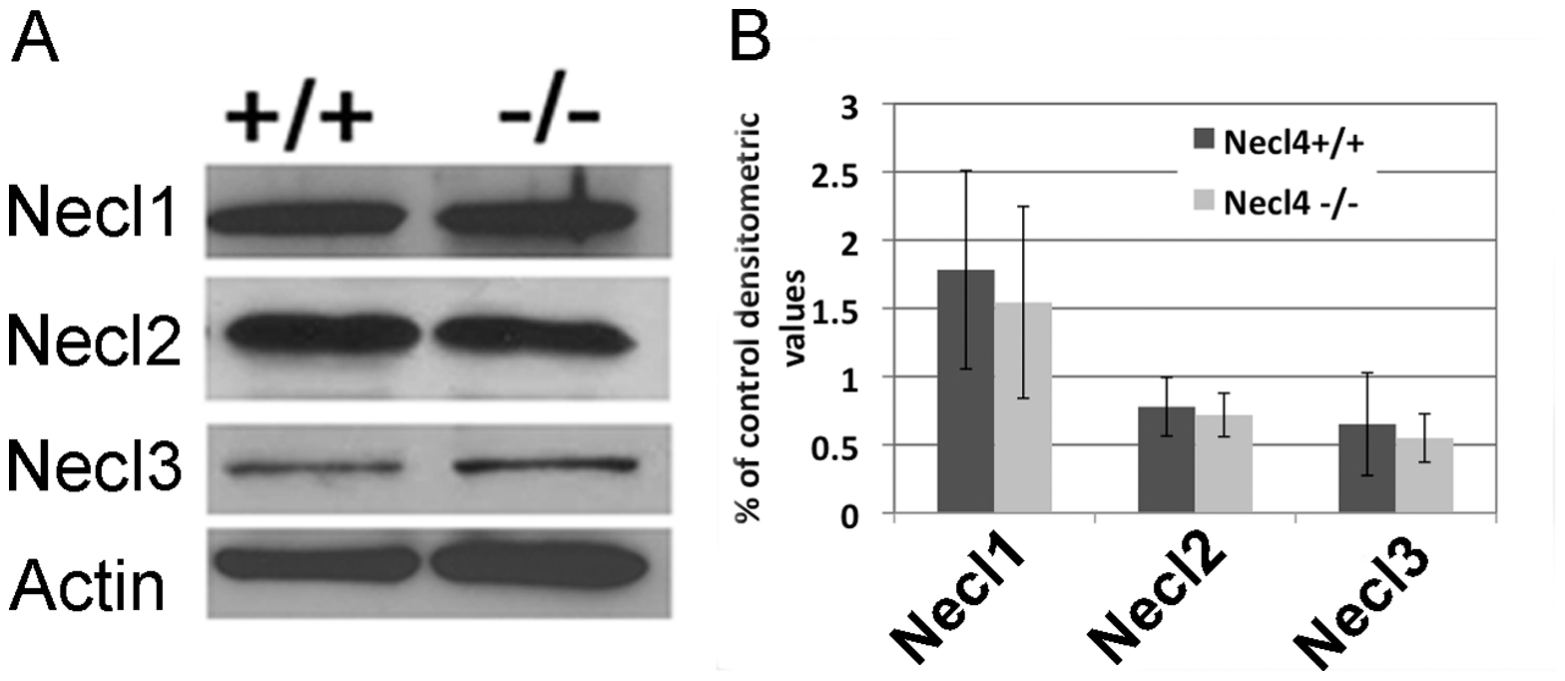
The expression level of Necl1, Necl2 and Necl3 proteins was not changed in the CNS of *Necl4* mutants. **A.** Western immuneblotting of brain tissues (from P10 wild-type and Necl-4 homozygous mice) with anti-Necl-1, anti-Necl-2 and anti-Necl-3 antibodies. β-actin was the internal control. **B.** Statistical analysis on the relative expression level of Necl1, Necl2 and Necl3 with Student's t-test (n = 3. Necl1, *p* = 0.70. Necl2, *p* = 0.72. Necl3, *p* = 0.69). Error bar, standard deviation.

The lack of apparent phenotype in *Necl-4* mutant animals raised the possibility of potential functional redundancy between Necl-4 and other Necl proteins. Therefore, we examined the expression of Necl1-3 with ISH and western blot (Figure S2, Figure S3 and Figure 7). We had described earlier that Necl1 is only expressed by neurons in the CNS [16]. Our ISH results suggested that Necl2 was also robustly expressed in the gray matter of the spinal cord from E16.5 to P0. However, its expression was down-regulated soon after birth. At P30, little Necl2 staining was detected in the gray matter (Figure S2). Unlike other *Necl* genes, *Necl-3* only had weak expression in the gray matter of the spinal cord at embryonic stages, and no expression was detectable by ISH after birth (Figure S3). Thus, both Necl-2 and Necl-3 are not significantly expressed by oligodendrocytes at all stages. Western blotting results confirmed their relative expression levels in the CNS, and revealed that the expression of Necl-1, Necl-2 and Necl-3 was not significantly altered in the *Necl-4* mutants (Figure 7. n = 3. Necl1, *p* = 0.70. Necl2, *p* = 0.72. Necl3, *p* = 0.69). These results suggested that *Necl-4* did not cross regulate the expression of other members of the *Necl* family, and the loss of *Necl-4* in oligodendrocytes was unlikely to be compensated by the up-regulation of *Necl-2/3*.

## Discussion

The reciprocal communication between neurons and glia plays a critical role in the control of myelination process [21]. Previous studies described that *Necl-4* is expressed by PNS neurons and Schwann cells, and mediates axonal myelination by heterophilic binding to its axonal partner *Necl-1* [6], [7]. In this study, we systematically analyzed *Necl-4* expression in the developing CNS and found that *Necl-4* expression was initially detected in gray matter neurons, but later up-regulated in mature myelinating oligodendrocytes in the white matter (Figure 1–2) at early postnatal stages.

The up-regulation of *Necl-4* in oligodendrocytes is spatiotemporally correlated with the myelination events in the spinal cord and the brain. Myelination of axons by oligodendrocytes in the CNS primarily starts at neonatal stages, first in the spinal cord and later in the brain, following a rostral-to-caudal order in the spinal cord or a caudal-to-rostral sequence in the brain [5]. In the spinal cord, *Necl-4* started to be transcribed in differentiated oligodendrocytes at perinatal stages when myelination process commences, and reached the maximum at the peak time of myelin formation (from P7 to P15) in the spinal cord [4], followed by gradual down-regulation thereafter (Figure 1). A similar temporal correlation was also noticed between *Necl-4* expression and axonal myelination in the cerebellum and the forebrain (Figure 4). Together, these expression studies provided circumferential evidence for its possible involvement in mediating axon-glia interactions and promoting axonal myelination in the CNS.

Surprisingly, our genetic analyses demonstrated that disruption of *Necl-4* gene did not cause apparent developmental defects in the CNS. The *Necl-4* null mutant mice were viable and fertile, and did not display noticeable motor disorders. The normal expression pattern of mature oligodendrocyte markers in the mutant spinal cords at neonatal stages suggested that *Necl-4* is not required for oligodendrocyte differentiation and maturation (Figure 6A–E). The number and percentage of myelinated axons in spinal cord and optic nerve (Figure 6H, Figure S1 and data not shown) were similar in both controls and *Necl-4* null mutants. These results suggested that axonal myelination proceeded in a comparable pace in both mutants and the controls.

Contrary to the previous *in vitro* studies [6], [7], *Necl-4* homozygous mutants and their wild type littermates displayed similar pattern of myelination and thickness of myelin sheaths in the sciatic nerve (Figure 6I–K, Figure S1B), indicating that *Necl-4* does not play an essential role for developmental myelination in the PNS as well. Expression analyses did not support the idea of functional compensation by other Necl proteins, because *Necl-4* is the only Necl protein whose expression is upregulated in myelinating oligodendrocytes (Figure 1, Figure S2, S3), and disruption of Necl-4 did not significantly change the expression level of other Necl proteins in the CNS and the PNS (Figure 7 and data not shown). One plausible explanation for the discrepancy between *in vitro* experiments and *in vivo* observations is that the initiation of axonal myelination may also involve interactions between other cell adhesion molecules such as laminins and integrins [22]–[25] and this interaction might be disrupted in dissociate culture and therefore can not compensate for the loss of Necl-1/Necl-4 interaction. Further studies with compound mutants of *Necl-4* and other adhesion proteins could delineate the role of various cell adhesion molecules in myelination in the developing nervous system.

## Supporting Information

Figure S1 **A.** The density of axons in the spinal cord at P7. n = 3. Student's t-test, *p* = 0.81. **B.** The average number of myelinated axons in the sciatic nerves at P7. n = 4, Student's t-test, *p* = 0.50. **C.** The average perimeter of axons in the sciatic nerves at P7. n = 4, Student's t-test, *p* = 0.80. Error bar, standard deviation. SC, spinal cord. SN, sciatic nerve.
(TIF)

Figure S2 Expression of Necl2 in the developing spinal cord. **A–F:** Spinal cord sections from E16.5, E18.5, P0, P7, P15 and P30 were subjected to ISH with *Necl-2* riboprobe. There was no apparent *Necl-2* expression in the white matter region at all stages examined.
(TIF)

Figure S3 Expression of Necl3 in the developing spinal cord. **A–H:** Spinal cord sections from E16.5, E18.5, P0, P4, P7, P15, P30 and P64 were subjected to ISH with *Necl-3* riboprobe.
(TIF)
