# Supplementary figures and images for: Necl-4/SynCAM-4 Is Expressed in Myelinating Oligodendrocytes but Not Required for Axonal Myelination
Source: PLoS One. 2013 May 20;8(5):e64264. doi: 10.1371/journal.pone.0064264 (PMC3659047; doi:10.1371/journal.pone.0064264)

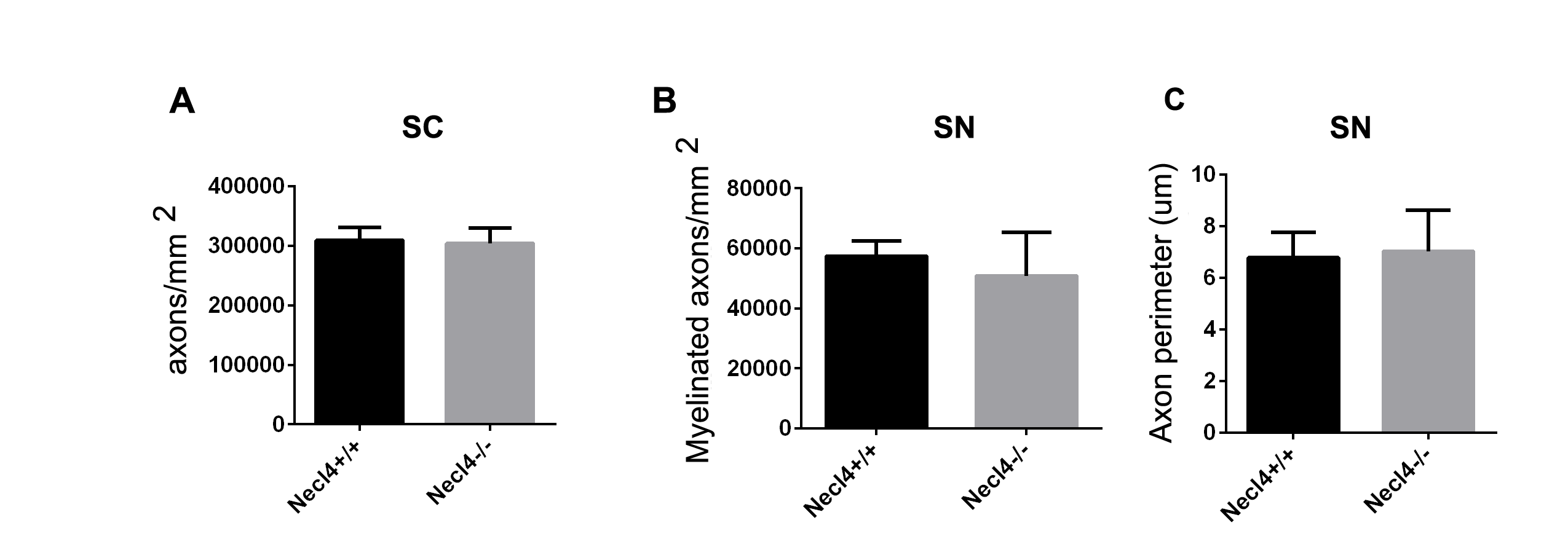

Supplement: Figure S1 — A. The density of axons in the spinal cord at P7. n = 3. Student's t-test, p = 0.81. B. The average number of myelinated axons in the sciatic nerves at P7. n = 4, Student's t-test, p = 0.50. C. The average perimeter of axons in the sciatic nerves at P7. n = 4, Student's t-test, p = 0.80. Error bar, standard deviation. SC, spinal cord. SN, sciatic nerve. (TIF) [file pone.0064264.s001.tif]

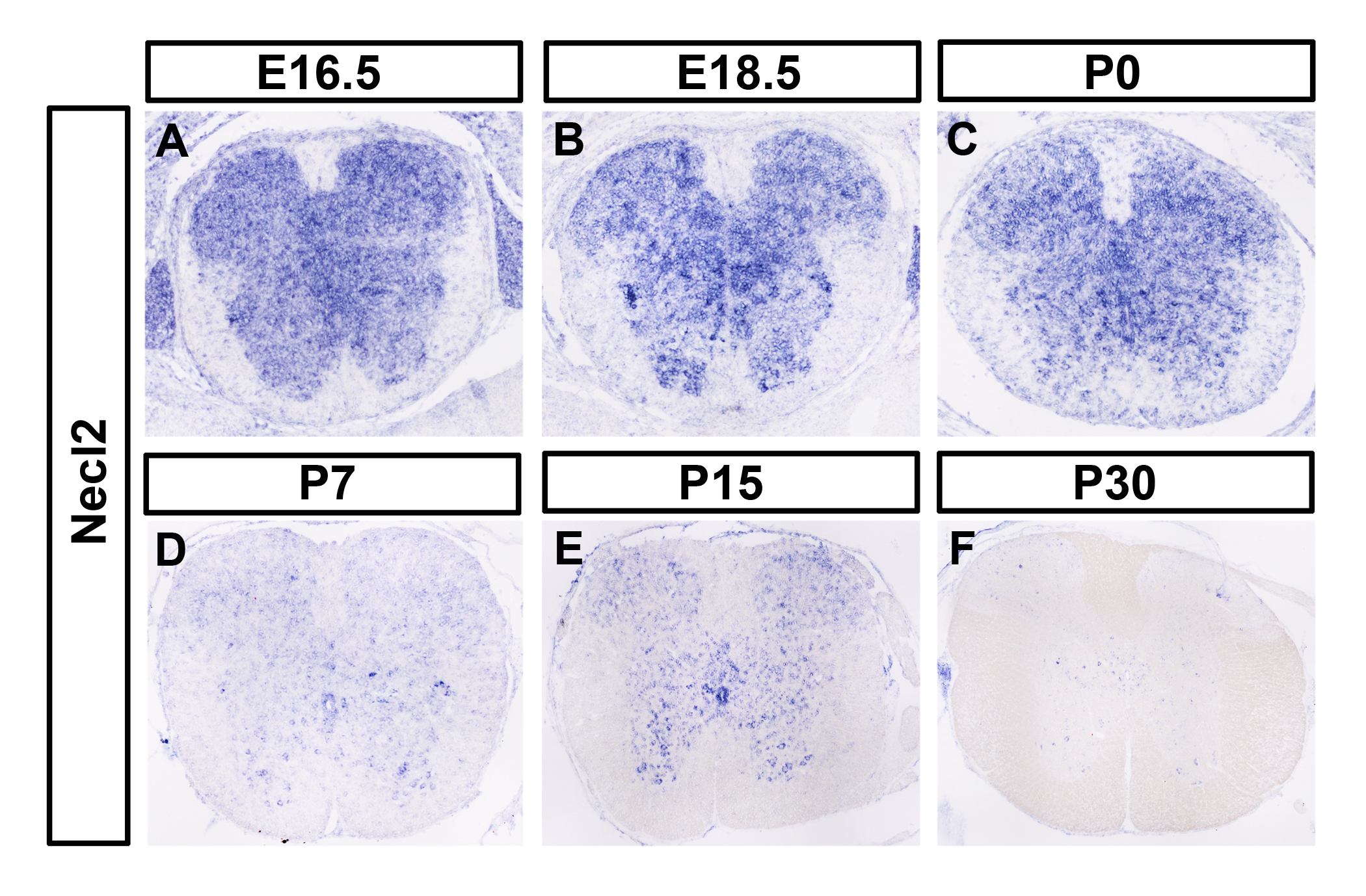

Supplement: Figure S2 — Expression of Necl2 in the developing spinal cord. A–F: Spinal cord sections from E16.5, E18.5, P0, P7, P15 and P30 were subjected to ISH with Necl-2 riboprobe. There was no apparent Necl-2 expression in the white matter region at all stages examined. (TIF) [file pone.0064264.s002.tif]

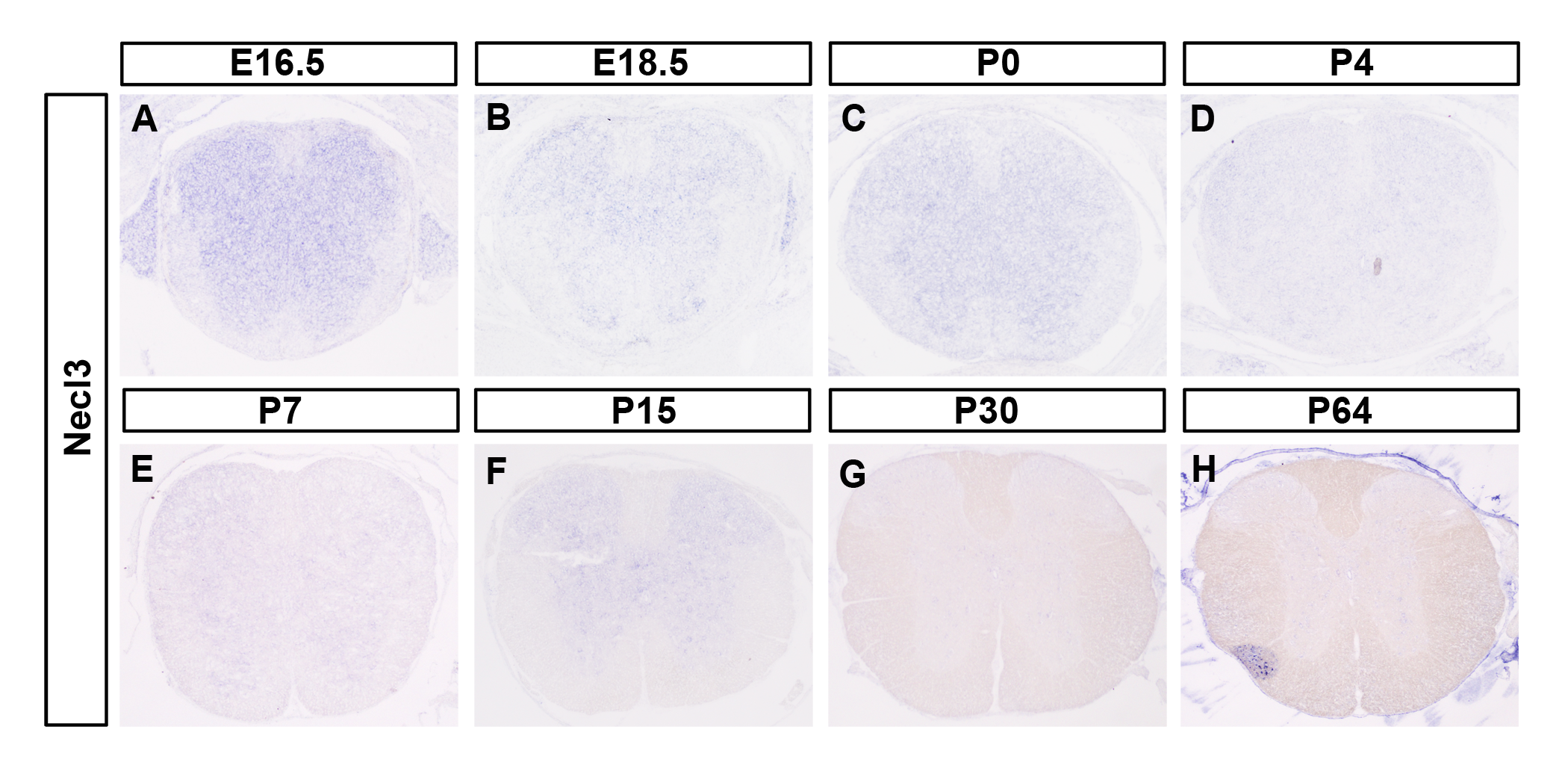

Supplement: Figure S3 — Expression of Necl3 in the developing spinal cord. A–H: Spinal cord sections from E16.5, E18.5, P0, P4, P7, P15, P30 and P64 were subjected to ISH with Necl-3 riboprobe. (TIF) [file pone.0064264.s003.tif]
